# Quality of life and prosocial or antisocial coping with resource deprivation: A cross-sectional study of people at risk of social exclusion

**DOI:** 10.1371/journal.pone.0275234

**Published:** 2022-09-28

**Authors:** Joanna Chwaszcz, Rafał P. Bartczuk, Iwona Niewiadomska, Patrycja Sławska-Jaroszewska

**Affiliations:** Institute of Psychology, The John Paul II Catholic University of Lublin, Lublin, Poland; Universitat de Valencia, SPAIN

## Abstract

**(1) Background:**

This paper presents a study based on Stevan Hobfoll’s conservation of resources theory and deals specifically with resources and coping as predictors of the quality of life of persons threatened by social exclusion. They have no access to public psychosocial resources, the acquisition and accumulation of which are closely linked to the perceived quality of life.

**(2) Method:**

A cross-sectional self-reported questionnaire study. A sample of 1,074 individuals from various groups at risk of exclusion was surveyed using the Conservation of Resources-Evaluation Questionnaire, the Strategic Approach to Coping Scale, and the WHO Quality of Life Questionnaire. The research was done among people supported by Polish non-governmental organizations operating nationwide. Structural equation modeling was used to test mediational hypotheses in the analysis.

**(3) Results:**

The significant variables that determined the relationship between resource gains and losses and the quality of life included active–passive and prosocial–antisocial coping strategies. The results were generally robust, but the level of education moderated the relationship between active antisocial coping and quality of life.

**(4) Conclusions:**

The obtained dependencies are consistent with Hobfoll’s approach, confirming its usefulness. Prosocial coping mediates the effect of resource gain on the increase in quality of life and the effect of resource loss on the decline in quality of life, strengthening the former and weakening the latter.

## Introduction

The conservation of resources theory (COR) emerged in the late 1980s from psychosocial theories of stress and motivation and provided a basis for understanding processes involved in experiencing, coping with, and resilience to chronic and traumatic stress [1–3]. Classic approaches to stress recognized that personal resources (e.g., self-efficacy) and social resources (e.g., emotional support) protect against negative impact of hardships in life [4–6]. COR complements the previous theories by recognizing stress as a response to a situation that exceeds the capabilities of the individual’s resources, threatens them, or leads to their exhaustion. According to COR, individuals strive to gain, preserve, and protect what they value, so stress occurs in situations involving a threat of loss or an actual loss of resources, or in situations where an investment of resources does not generate the intended effects [7]. COR is an ecological and multi-level approach, as it places the individual’s behaviour in the context of family, community, and culture. Hobfoll’s assumptions have been supported by both theoretical and empirical work and are applied in many areas [7–12].

### COR approach to coping

Resource distribution and the experience of stress force the individual to initiate coping strategies. In COR, coping is understood as using existing resources in situations perceived as threatening to protect owned resources or to gain new resources [3]. In particular, COR highlights the use of communal resources. The culture in which a person operates is linked to the specific stress situations, resources, and society-preferred strategies. Therefore, apart from the traditional distinction between active and passive strategies, COR draws attention to the social dimension of coping (antisocial vs prosocial) and the dimension of directness vs indirectness in the approach to solving problems that reflect intra- and inter-cultural diversity [13–16]. Individualistic cultures value personal reduction of mental discomfort, which involves taking care of one’s own needs more than the needs of others. Thus, the importance of active, individual (“asocial” in COR terminology) and direct action is stressed. On the other hand, collectivist cultures see a broadly understood community, or communal support, as a value in solving stressful situations, promoting active, prosocial, and indirect action [3].

Dunahoo, Hobfoll et al. [13] distinguished nine coping strategies: assertive action, avoidance, seeking social support, cautious, social joining, instinctive action, aggressive action, antisocial action, and manipulative (indirect) action. These strategies were grouped into three second-order categories: passive-active, active-prosocial, and active-antisocial. An up-to-date scoping review demonstrated that while some first-level factors are more culturally stable than others, the second-order structure is generally interculturally stable [17].

The effectiveness of coping strategies depends on the optimal use of resources and making decisions in line with one’s values and the environmental context [18, 19]. How individuals manage their resources and what coping strategies they prefer affects their psychophysical and social functioning. Numerous studies have shown that active coping and social support seeking promote a higher level of well-being than avoidant and antisocial strategies [20–22]. Polish studies among young people with mild intellectual disability confirmeda positive correlation between prosocial coping and life satisfaction as well as a negative correlation between antisocial coping and life satisfaction [23]. Using maladaptative coping strategies can have health, psychological, and social consequences. In a Spanish study among multiple sclerosis patients [24], it was demonstrated that the patients, compared to the group of healthy people, used more strategies such as avoidance and instinctive action and showed fewer social joining attitudes.

According to the COR, resources and their growth are associated with a higher quality of life, and their loss with a lower quality of life. Those capable of creating, accumulating, and maintaining resources are less likely to lose them and thus more likely to experience a sense of success and gain, which translates into life satisfaction [3, 22, 25]. Research has also confirmed that a gain in personal resources in the group of those at risk of social exclusion can predict increased life satisfaction over time [26].

### Quality of life and resources in those at risk of exclusion

From the psychological point of view, individuals’ quality of life is determined by their subjective assessment of experiences and objective factors. According to WHO experts, quality of life should be assessed through the person’s perceptions of their position in life, cultural context, values, interests, goals, expectations, and standards [27]. The perceived quality of life is affected by multiple factors, including interpersonal relations, life environment, psychological and physical state, and macroeconomic resources [28, 29].

People at risk of social exclusion experience a low quality of life in a particular way [30–34]. Marginalization consists in excluding an individual from the community, either physically or emotionally, through interpersonal rejection, ostracism, or various types of discrimination. The threat of exclusion entails limited access to material, institutional, and social resources, poor health, often destructive environment, and low-quality interpersonal relations [35–37]. For example, those with substance addiction often experience rejection from their loved ones and social stigma, which reduces their perceived quality of life [26]. Also, the long-term exclusion is accompanied by an expectation of rejection in everyday interactions, experiencing learned helplessness, and emotional difficulties [38].

COR provides conclusions that can be applied to those at risk of social exclusion. First, they are more prone to loss of resources than the socially included. Second, losses are especially severe for them [3, 39]. Third, resource loss is especially dangerous due to the lack of resource reserves, a result of which the necessary resource investments are not adequately secured and trigger a loss spiral. A study of alcohol addicts during therapy showed that those with initially fewer resources were more likely to discontinue treatment and return to their destructive lifestyle [26, 40]. A resource-rich social environment tends to prevent the use of resources by disadvantaged groups. Individuals who experience a resource loss attributed to society may try to distance themselves from stress by engaging in passive and asocial coping, the consequences of which may be further resource loss. This kind of process was confirmed by the study by Hassanbeigi et al. [41] among opium addicts. Over two years, the respondents experienced highly stressful situations much more often than healthy individuals and used coping strategies focused on emotions and avoidance more often, making their psychophysical state deteriorate. A study among multiple sclerosis patients showed similar dependencies [24].

The choice of coping strategies by those at risk of exclusion depends not only on their characteristics, including the currently available or recently gained or lost resources, but is also determined by the availability of resources for their group in their specific environment. Coping strategies may also change as a result of changes in the individual structure of resources or their availability. For example, a qualitative study conducted among women displaced from conflict-stricken areas in Georgia showed that they activated various coping strategies when experiencing a loss spiral [21]. In studies of various Polish groups of people with an offence record, problem-orientation combined with prosocial strategies were characterized by higher adaptation and well-being in comparison to those subjects who use passive, asocial, or antisocial strategies [22, 42].

The COR emphasizes that depending on the type of hardship, the same coping strategies may bring different results: defusing a difficult situation, adapting to it, or incurring a cost [43, 44]. This is illustrated by a situation where excessive use of avoidance strategies by addicts could have contributed to the development of a dependence syndrome. In contrast, moderate avoidance strategies may help addicts initially maintain abstinence at the initial stage of recovery [45].

### Socio-demographic factors in resources distribution and coping

Resource distribution, coping, and quality of life are influenced by socio-demographic factors such as gender, age, education, and residence. Numerous studies have highlighted gender differences in preferred coping strategies [46–48]. According to COR, gender differences in coping are structural and result from diversified access to resources and fulfilling different social roles [14, 15]. Developing a multi-axial model of coping, considering the community context, Hobfoll [3] showed no gender differences in coping, although he noticed some regularities regarding preferences of coping strategies. Hobfoll also observed age differences in the choice of a coping strategy. Time also plays a vital role in managing resources because some are available at specific stages of life [7]. Another factor, level of education, can be classified as a resource that can be exchanged for other resources, and therefore it can be directly related to the level of resources and the applied coping strategies [49]. Generally speaking, socio-demographic variables, reflecting the individual’s position in society, are related to social roles, possibilities of acquiring specific resources, and socially expected preference for coping strategies and determine the social policies and support addressed to people at risk of exclusion.

The goal of the present study was to explore the mechanisms predicting quality of life in people with limited access to resources. To achieve this, we envisaged two aims: (a) to examine the relationships between resources (interpreted as profit and loss) and quality of life; and (b) to check which of the socio-demographic variables (gender, age, education) can modify the interdependence of resources, stress coping strategies and quality of life. The analyses were made based on the COR approach: the quality of life of those at risk of marginalization depends on the distribution of resources and the coping strategies used [3, 22, 25]. The specific features of the situation of risk of marginalization include its long duration, which requires coping strategies leading to adaptation in a situation of chronic stress, and limited access to social resources, which hinders the use of prosocial strategies. Overall, we expect coping strategies to mediate between the experienced resource gains and losses and the quality of life of the respondents. In particular, we assume that resource gains will predict active and prosocial strategies, while resource losses–antisocial strategies. Thus, active and prosocial strategies will be associated with an increase in the quality of life, while antisocial strategies, with its decrease. In addition, some structural socio-demographic factors can influence general regularities, so we assumed relationships could be moderated by such socio-demographic variables as age, gender and education.

In particular, we expected that coping strategies would mediate between the experienced resource gains and losses and the quality of life of the respondents in such a way that resource gain would be a predictor of active and prosocial strategies, while resource losswould predict antisocial strategies; and that active and prosocial strategies would be associated with increased quality of life, the use of antisocial strategies, and with its decline.

## Materials and methods

### Subjects and procedure

Data for the present study were collected during a broader research project. The eligibility criteria for participation in the study required that at least one of the following conditions was met (now or in the past): an experience of prostitution, underage motherhood, a home for teen mothers, homelessness, prison, a correctional facility, an orphanage, alcoholism in the family, or substance abuse. All respondents used the support offered by non-governmental organizations (NGOs) in various forms in the last 12 months. Therefore, due to the conditions mentioned above and using the help, we concluded that these people meet the risk of social exclusion criteria. Some of the respondents stayed temporarily in the centers but functioned in their natural social environments while benefiting from the support of NGOs. The study excluded people living in closed facilities, in particular detained in prisons. The previous research showed that the relationship between coping and quality of life of prisoners is shaped in a different way than that of people enjoying freedom due to the unique experience of staying in a correctional facility [50, 51].

The study was carried out in assistance and re-socialization institutions across Poland, including homeless shelters, single mothers’ homes, municipal social welfare centres, crisis intervention centres, and outpatient therapeutic centres. Invitations were sent to support centres run by NGOs across using a list published by portal run by NGOs (www.ngo.pl). The research was done in centres whose principals gave consent. Next, verbal consent was obtained from the subjects. Surveys were run only among those who expressed their informed verbal consent to the research, in accordance with the Helsinki Declaration (the 2013 revision). This study was approved by the ethics committee of the Foundation for the Development of the Catholic University of Lublin (FRKUL/WSZW/KIN/05-15). Thirty trained interviewers carried out face-to-face surveys in designated institutions and the data was anonymized.

The study covered a total of 1,074 people. The demographics of the subjects were as follows: 57.7% (*n* = 620) were women at the mean age of 26.3 years (*SD* = 11.95), 34% (*n* = 265) lived in rural areas, 32.9% (*n* = 350) had primary, 17.2% (*n* = 183) vocational, 28.9% (*n* = 307) secondary, and 21% (*n* = 107) higher education.

Those who completed at least 80% of the items in each of the questionnaires were included in the analysis. There were 1,037 such respondents (response rate 96.6%). Excluded individuals did not differ in any socio-demographic variable from the rest of the respondents. The remaining missing responses to individual items were imputed using medians.

### Measures

Respondents completed an extended package of methods. The present analysis used scores from the Conservation of Resources–Evaluation (COR-E), the Strategic Approach to Coping Scale (SACS), the short version of the WHO Quality of Life Questionnaire (WHOQL-BREF), and a questionnaire to collect socio-demographic data.

#### Resource gain and loss

The Polish version of the COR-E questionnaire [52–54] was used to assess resources distribution. The questionnaire contains a list of 74 resources. When assessing each item, the respondent refers to each resource on a 5-point scale (from 1 = *Not at all* to 5 = *To a great degree*) in two categories: loss and gain. In the Polish adaptation a bi-factorial COR-E structure composed of the global resources factor and seven group factors was confirmed [52]. In the present sudy, the resources gain and resources loss scores were obtained using parceling [55]. Parceling removes the problem of item covariance caused by group factors [56], so the latent variable thus obtained can be understood as the gain and loss of the overall resource potential of an individual. In this study, Cronbach’s α was .96 for Gains and .98 for Losses.

#### Coping

The Polish version of the SACS [SACS-PL; 57] was used to measure coping strategies in line with COR assumptions. The SACS-PL is based on the situational version of the SACS developed by Monnier, Hobfoll et al. [16], which consists of 52 items with a 5-point response format ranging from 1 (= *Didn’t do this at all*) to 5 (= *Did this a lot*). A situational version of the method is more relevant than dispositional for studying those in different situations related to social exclusion risk [58]. When answering questions, respondents referred to the general difficult life situation they had found themselves in recently. An adaptation study showed that the SACS-PL has six subscales that form three second-order factors [57]. In the present study, second-order latent factors were modeled using the subscales’ scores from the first-order structure. The latent factor of avoidance coping was modeled using parceling to avoid using one indicator per factor. The following Cronbach’s alphas were obtained on first-order subscales: Dominating action.82, Assertive action .76, Avoidance .82, Social support seeking .78, Considerate action .58, and Instinctive action .43.

#### Quality of life

Quality of life was measured with the WHO Quality of Life-BREF (WHOQOL-BREF), an abbreviated generic quality of life scale developed through the World Health Organization [59, 60]. It is an international cross-culturally comparable quality of life assessment instrument simultaneously developed in 15 countries. The WHOQOL-BREF comprised 26 items, which measure the following broad domains: physical health, psychological health, social relationships, and environment [61]. In the present study, quality of life was modelled as the latent variable with subscale scores as indicators. In this study, the following reliability coefficients were obtained: Physical domain .77, Psychological domain .77, Social relationships domain .69, and Environment Domain–.75.

#### Socio-demographic variables

In the present study, we used questions about age (open-ended question), gender, education, and place of residence. Variables were dichotomized before analysis.

### Statistical analyses

The research hypotheses were tested employing structural equation modeling (SEM), using robust maximum likelihood implemented on the lavaan [62] and semTools [63] packages in the R statistical environment [64]. The random item-to-parcel allocation procedure [65, 66] was used, and 100 random assignments have been generated for each of the parceled methods. Data sets obtained in this way was analyzed as multiply imputed data set. Reported parameters are pooled point and confidences intervals estimates, following Rubin’s [67] rules. To assess model fit, we examined absolute (χ^2^, *SRMR*), incremental (*CFI*, *TLI*), and parsimony-adjusted (*RMSEA*) fit indices [68]. Given our data, sample size, and estimation procedures, we set cutoff values of >.90 for both the *TLI* and *CFI*, ≤.06 for *RMSEA*, and ≤.08 for the upper value of its 90% CI, < .08 for *SRMR* [69, 70]. Robust confidence intervals for direct and indirect effects were obtained using Monte Carlo [71]. Assessment of effect sizes were done following Peterson and Brown [72] rules. Due to the large sample size, we assumed that effects would have to be interpreted using a more stringent *p*-value (*p* ≤ .01).

## Results

Basic descriptive statistics and zero-order correlation coefficients calculated for the variables in the study are attached in the S1 Table. Generally, it can be said that the pattern of correlations showed links between resource distribution, coping strategies, and quality of life, in line with the assumptions of the COR theory.

In the main analysis, we tested the hypothesis that coping strategies mediate the effects of resource loss and gain on the quality of life. For this purpose, we built SEM, which included resource loss and gain as endogenous variables, coping strategies as mediators, and quality of life as the explained variable. Resource loss and gain latent variables were built using 4 each randomly generated parcels. Three latent variables were built to model coping strategies, in line with the second-level analysis of the SACS subscales for Polish culture [57]: (1) prosocial coping composed of social support seeking, considerate action, and assertive action; (2) antisocial coping composed of dominating action, instinctive action, and cross-loaded assertive action, and (3) avoidance, originally composed of one indicator, was modeled as composed of two parcels. The latent variable quality of life was constructed from the four quality of life domains included in the WHOQOL-BREF: physical, psychological, social relationships, and environment. Then, we tested the measurement model, in which the relationships between latent variables were modeled as covariance. The model achieved good fit indices: χ^2^(136) = 63.071, *p* = 1.00; *CFI* = 1.00, *TLI* = 1.00, *RMSEA* = 0.0 (*95%CI*[0.0, 0.0]); *SRMR* = .034.

Error-free correlations of latent variables from the Measurement Model are presented in Table 1.

**Table 1:** Error-free pooled correlations of measurement model with 95% confidence intervals (N = 1037).

Variable	1	2	3	4	5
1. Loss of resources					
2. Gain of resources	0.10**				
	[0.03, 0.16]				
3. Avoidance coping	0.17***	-0.06			
	[0.10, 0.23]	[-0.12, 0.01]			
4. Antisocial coping	0.17***	0.11***	0.68***		
	[0.11, 0.23]	[0.05, 0.17]	[0.63, 0.72]		
5. Prosocial coping	-0.07*	0.37***	0.26***	0.37***	
	[-0.13, -0.01]	[0.31, 0.42]	[0.17, 0.34]	[0.31, 0.42]	
6. Quality of life	-0.34***	0.50***	-0.15***	-0.01	0.46***
	[-0.39, -0.29]	[0.45, 0.55]	[-0.21, -0.08]	[-0.07, 0.05]	[0.41, 0.51]

Note. ***p < .001

**p < .01

*p < .05.

### Mediation model

**Fig 1:**
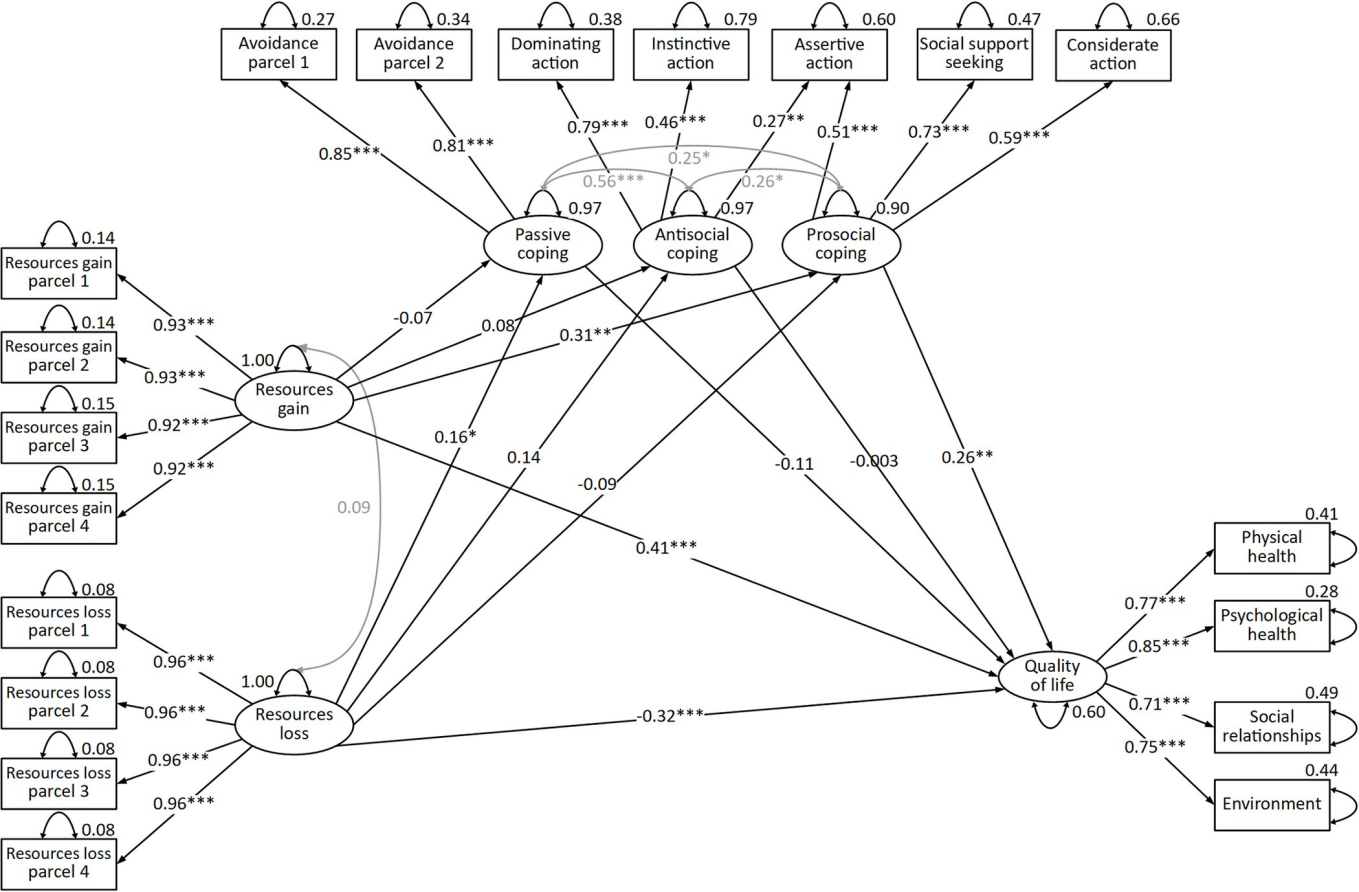
Structural equation mediation model of resources gain and loss, coping, and quality of life (*N * =  1037). *p < .05, **p < .01, ***p < .001.

In the second step, we tested the mediation model (Fig 1), which also achieved satisfactory fit indices: χ^2^(136) = 64.241, *p* = 1; *CFI* = 1, *TLI* = 1, *RMSEA* = .0 (*95%CI*[.0, .0]); *SRMR* = .034.

The model accounted for 40% (pooled *R*^*2*^) of the variance in quality of life. The direct effects of resource gain and loss on quality of life were significant and large (Table 2). Resource loss reduced and resource gain increased the quality of life. Total indirect effects of both main explanatory variables turned out to be significant. Both of these effects have signs consistently with the direct effect, and the effect of gain is small, while loss is minimal. Indirect effects accounted for 22% of the total gain effect and 13% of the total loss effect on the quality of life. Resource gains increased quality of life by increasing prosocial coping, which in turn was positively correlated with quality of life.

**Table 2:** Total, direct, and indirect effects of resources gain and loss on quality of life through coping strategies (*N* = 1037).

Main predictor	Effect	B	95%CI[LL, UL]	β
Gain of resources	Total	0.64	[0.549, 0.735]	0.407
	Direct	0.53	[0.441, 0.614]	0.496
	Total Indirect	0.12	[0.066, 0.169]	0.089
	Indirect via PAC	0.01	[-0.001, 0.025]	0.007
	Indirect via PSC	0.11	[0.061, 0.156]	0.081
	Indirect via ASC	0.00	[-0.013, 0.012]	0.000
Loss of resources	Total	-0.47	[-0.557, -0.389]	-0.324
	Direct	-0.42	[-0.500, -0.336]	-0.365
	Total Indirect	-0.05	[-0.090, -0.022]	-0.042
	Indirect via PAC	-0.02	[-0.048, -0.004]	-0.018
	Indirect via PSC	-0.03	[-0.060, -0.005]	-0.024
	Indirect via ASC	0.00	[-0.020, 0.019]	0.000

Note. *B* = parameter, β = standardized parameter; CI = confidence interval; *LL* = lower limit; *UL* = upper limit. PAC = Avoidant coping, PSC = Prosocial coping, ASC = Antisocial coping.

### Moderation of model by socio-demographic variables

We then checked whether the socio-demographic variables, i.e., gender, age, place of origin, and education, moderate the identified relationships. For this purpose, a series of multi-group structural models were built, one for each moderating variable. First, the measurement invariance was checked up to the level of the factor for the groups distinguished based on the moderator level. To test invariance we first fitted the measurement model leaving all factor loadings and item intercepts free to vary for each group (configural model: CM). In the next step we tested for metric invariance to examine whether the factor loadings are equivalent across the groups by constraining the factor loadings to be equivalent across groups (loadings model: LM), while still allowing the item intercepts to vary freely as before. The difference between the *CFI*s of CM and LM was used as the criterion. According to Cheung and Rensvold [73], invariance is likely untenable when the difference is greater than 0.01. After loading invariance was determined, we compared another two multi-group structural models: the unconstrained model, with fixed loading sizes equal in the tested groups, and the regression model with additionally fixed regression weights equal in both groups. If no differences were found between these models, it was assumed that the tested variable did not act as a moderator.

#### Gender

Two groups were distinguished: women (*n* = 597) and men (*n* = 440). Invariance analysis of the measurement model showed that the measurement could be considered equal on the level of intercepts. The comparison of mediation models with unconstrained and constrained regression weights showed no differences (F(11, 858154) = 1.26; *p* = .239; Δ*CFI* < 0.001).

#### Age

Two groups were distinguished: under 25 years of age (*n* = 682) and at least 25 years of age (*n* = 355). Invariance analysis of the measurement model showed that the measurement could be considered equal on the loadings level. The comparison of mediation models with unconstrained and constrained regression weights showed no differences (F(11, 2439682) = 0.972; *p* = .469; Δ*CFI* < 0.001).

#### Place of residence

Two groups were distinguished: rural residents (*n* = 220) and city dwellers (*n* = 817). Invariance analysis of the measurement model showed that the measurement could be considered equal on the level of means. The comparison of mediation models with unconstrained and constrained regression weights showed no differences (χ^2^(11) = 7.98; *p* = .715; Δ*CFI* < 0.001).

#### Education

Two groups were compared: those with primary or vocational education (*n* = 515) and those with at least secondary education (*n* = 511). Invariance analysis of the measurement model showed that the measurement could be considered equal on the loadings level. The comparison of mediation models with unconstrained and constrained regression weights showed differences (χ^2^(11) = 20.63; *p* = .037; Δ*CFI* = 0.003). In a search for partial invariance, the model obtained after the unconstraint of quality of life regression on antisocial coping showed no differences against the unconstrained model (χ^2^(10) = 13.59; *p* = 0.193; Δ*CFI* = 0.001) at the level of loadings. In less educated people, the antisocial coping effect on quality of life was negative (*B* = -0.411, β = -0.11, *p* = .047), while in those better educated it was positive but insignificant (*B* = 0.305, β = 0.07, *p* = .194), so in the group with less education antisocial coping worsened quality of life. In comparison, in the better educated group it did not.

## Discussion

The present study aimed to apply COR to explain the quality of life of those in groups at risk of social exclusion The results of the analyses showed that resource gains best explain quality of life and prosocial coping. In turn, prosocial coping is associated with quality of life. The dependencies we found are consistent with the second COR principle formulated by Hobfoll, which is referred to as a resource acquisition rule. Essentially, it says that people need to invest in resources in order to be protected against losing them, to replenish them after a loss, or gain more assets. The resource investment rule implies several important things. The initial resource gain contributes to the creation of further gains, generating spirals of resource gains. Their successive cycles become more likely due to the increasing availability of assets and their possibility of being further invested. It is precisely through resource investment that people not only reactively deal with stress but also proactively prevent its occurrence, which ultimately increases quality of life and the psychological resilience to stress [1–3, 7]. Persons who have more resources are less likely to be affected by stressful situations because they have the means to meet the challenges generated by stressful events. A higher level of internal resources also contributes to better utilization of internal resources (e.g., emotional and/or instrumental support). Having more resources is correlated with a lower level of negative effects of stress [74–77]. Lee et al. [78] arrived at similar results when investigating quality of life, socioeconomic resources, and social support and depression as mediators in alcohol-dependent patients. Socioeconomic resources proved to be related to quality of life both directly and also through the mediating role of social support. In a study by Lara et al. [79], a correlation between psychosocial resources and happiness felt by the elderly was confirmed. These authors also highlighted the role of social support as a mediator between satisfaction with physical health and a sense of happiness. Myck et al. [80] highlighted the importance of individual resources for the well-being of the elderly. The most potent activator of resource gains in our study was prosocial coping. Resource gains are therefore associated with active action [20–22].

Prosocial coping increased quality of life, while antisocial coping was not associated with quality of life in the entire study sample. However, it had a negative effect on quality of life only in those with low (primary and vocational) education. Antisocial coping includes strategies such as dominating action, instinctive action, and assertive action. The latter is also a component of prosocial action. Therefore, it can be hypothesized that those with secondary and higher education who are in a situation of social exclusion use antisocial strategies with the awareness of their choice as appropriate to the situation, possibly also in order to emphasize their autonomy towards the environment of the excluded, with whom they do not necessarily identify [81]. Such choices may, in line with the concept of self-regulation, even improve an individual’s well-being [23]. A cautious conclusion can also be made that the level of education is a protective factor (moderator) in terms of the sense of well-being. Such results suggest the need for further research in this area.

Resource loss had a negative correlation with the quality of life. It strengthened passive coping by avoiding a difficult situation. Lee et al. [78] reached similar conclusions and pointed to the relationship between the lack of socioeconomic resources and addiction (substance use as an avoidant coping strategy). People who have many resources are less affected by the negative influence of loss, while those who use fewer resources are more exposed to further losses. Therefore, a shortage of resources not only causes an exposure to risk but also that an initial loss leads to others [3, 7]. Upon an initial loss, low-resource persons are at risk of losing resources, and another loss gives rise to a cycle that further depletes resources and thus the impact of the loss is faster and more severe. Defensive attitudes resulting from resource deficits can have various manifestations: people may demonstrate aggression, irrationality, passivity, avoidance, and substance use [3, 7].

In conclusion, it should be emphasized once again that the most important mediator between resource gains and quality of life is prosocial coping. It boosts the positive prominence of the relationship between resource gains and quality of life. In recent decades, numerous studies have confirmed a positive relationship between prosociality and well-being. In a recent meta-analysis of over 200 studies on this relationship, Hui et al. [82] demonstrated a positive effect of prosociality on medium-sized well-being. In particular, the impact of prosociality on eudaimonic well-being was stronger than on hedonic well-being. Prosociality was most strongly associated with psychological functioning, showing a more modest association with a mental disability or physical health. The authors of that study suggested that moderators mask the covariation of prosociality and well-being. Such a moderator may be the situation of social inclusion or exclusion. However, this hypothesis requires further research.

### Limitations of the study

The study’s primary methodological limitation is its cross-sectional character, limiting the possibility of drawing conclusions about the causal nature of the relationships found, in particular mediation relationships. Further research work should be directed towards the application of the longitudinal model.

## Conclusions

Our research confirms the relationship between resource gains and loss and perceived quality of life. It also indicates the significant role of active and prosocial coping, which positively correlates with the quality of life and positively mediates the relationship between resource gains and quality of life. Passive copping, in turn, is associated with resource loss and decreases the sense of quality of life In turn, education moderates the relationship between active antisocial coping and quality of life. In those with less education (primary and vocational), antisocial coping reduces quality of life, while in people with at least secondary education it does not. These data contribute to global literature with an understanding of the factors and mechanisms that affect the quality of life in disadvantaged people. The results we obtained offer a prospect for further research, especially on the mediating role of active prosocial in shaping the well-being of low and high-resource individuals. The results obtained in the study are of great practical importance. After all, quality of life factors are among the variables that can be influenced by psychological, psychotherapeutic, and readaptation efforts. The results of our research confirmed that the distribution of resources plays a crucial role in coping with stress and the psychological effects of using specific coping methods [19]. Strengthening the personal resources of people at risk of exclusion is a critical element of social re-adaptation [83]. However, sourcing resources requires an environment in which those resources are available—both human and material resources. Therefore, the social policy at the national, local, and community levels should support creating such environments. Whereas inclusion efforts must be made for marginalized people, the following are of primary importance: strengthening personal and environmental resources and ensuring the presence of resources in the psychosocial space that can facilitate the gaining/multiplying of resources by marginalized communities. The most crucial factor in a positive social participation process is the strengthening of the active prosocial coping strategy.

## Supporting information

S1 Table Descriptive statistics and zero-order correlation coefficients calculated for the variables in the study. ^a^ 1 **=** women and 2 **=** men; ***p < .001, **p < .01, *p < .05.
(XLSX)
